# Concurrent active pulmonary tuberculosis and small cell lung cancer: diagnostic challenges and therapeutic insights from a case report

**DOI:** 10.3389/fonc.2025.1564686

**Published:** 2025-09-04

**Authors:** Meiling Sun, Huaijun Ji, Ailing Liu, Ning Xu

**Affiliations:** ^1^ Department of Respiratory and Critical Care Medicine, Weihai Municipal Hospital, Cheeloo College of Medicine, Shandong University, Weihai, Shandong, China; ^2^ Department of Thoracic Surgery, Weihai Municipal Hospital, Cheeloo College of Medicine, Shandong University, Weihai, Shandong, China

**Keywords:** pulmonary tuberculosis, Small cell lung cancer, diagnostic challenges, multidisciplinary collaboration, case report

## Abstract

The coexistence of active pulmonary tuberculosis (TB) and small cell lung cancer (SCLC) is an exceptionally rare clinical phenomenon, presenting significant diagnostic and therapeutic challenges due to overlapping symptoms, radiological findings, and drug interactions. We report the case of a 68-year-old male with a four-month diagnostic journey, involving persistent cough, exertional chest tightness, and multiple inconclusive bronchoscopic examinations. Active TB was confirmed via sputum smear tests identifying acid-fast bacilli, while SCLC was diagnosed later through a third bronchoscopy, supported by elevated progastrin-releasing peptide (ProGRP, 127.28 pg/mL). The patient received a two-month course of anti-TB therapy before initiating four cycles of etoposide-cisplatin chemotherapy, followed by chest radiotherapy. Anti-TB treatment was intermittently paused during chemotherapy cycles to minimize drug interactions, and the patient completed 11 months of therapy. Follow-up imaging showed partial resolution of the left upper lung lesion, with normalized tumor markers (ProGRP: 66.20 pg/mL). However, at 17 months, disease progression was noted with a metastatic lesion in the right lower lobe. This case underscores the complex interplay between TB-induced chronic inflammation and tumor progression, highlighting the need for early tumor marker testing, advanced imaging modalities such as PET-CT, and tailored therapeutic strategies. Multidisciplinary collaboration is critical for optimizing outcomes in such rare and challenging scenarios. Further research into the mechanistic links between TB and SCLC could improve early diagnosis, guide therapeutic decisions, and inform preventive strategies.

## Introduction

Small cell lung cancer (SCLC) is an aggressive neuroendocrine malignancy, accounting for approximately 15% of all lung cancers (1). It is characterized by rapid tumor growth, early metastasis, and poor prognosis despite initial responsiveness to chemotherapy and radiotherapy. In contrast, active pulmonary tuberculosis (TB) remains a global health concern, particularly in regions with a high disease burden (2). The coexistence of SCLC and active TB is exceptionally rare, presenting significant diagnostic and therapeutic challenges due to overlapping clinical and radiological features (3). Pulmonary nodules, consolidations, and lymphadenopathy, common to both conditions, often delay accurate diagnosis (4). Treatment is further complicated by the immunosuppressive effects of SCLC chemotherapy, which may exacerbate TB, and by drug-drug interactions between anti-TB and chemotherapeutic agents (5, 6).

Here, we report a rare case of concurrent SCLC and active pulmonary TB, diagnosed after multiple bronchoscopic procedures and a CT-guided biopsy. We also review the literature to explore the pathophysiological interplay between TB-induced chronic inflammation and tumorigenesis, and to discuss optimized diagnostic and therapeutic strategies for managing these dual conditions.

## Case presentation

**Figure 1:**
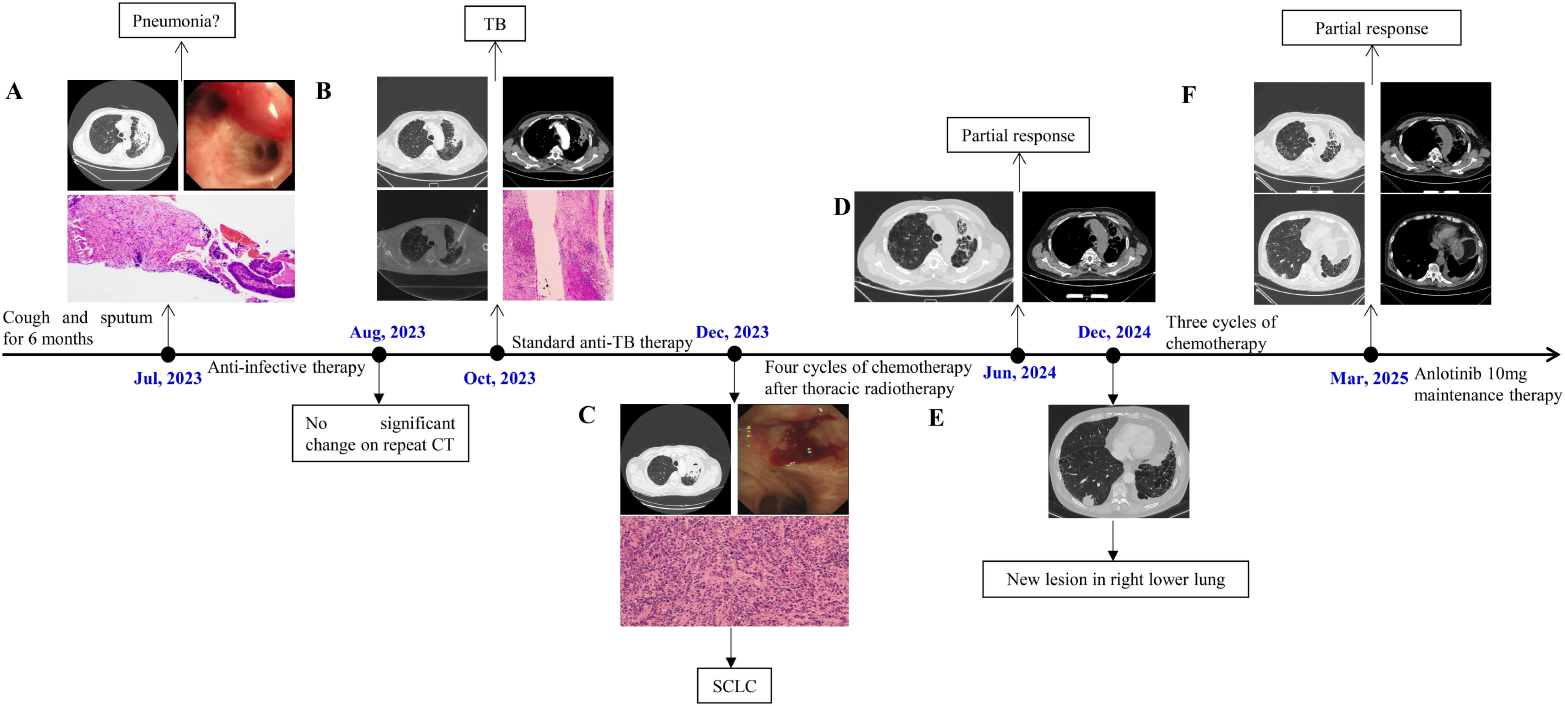
**(A)** CT showed a soft - tissue shadow in the left upper lobe of the lung (July 22, 2023); At the opening of the left upper lobe bronchus, a submucosal protrusion with hyperemia was observed; Pathological findings mild chronic inflammatory cell infiltration. **(B)** A contrast-enhanced chest CT revealed soft tissue shadows and uneven enhancement (October 5, 2023); CT-guided percutaneous lung biopsy; Pathological examination revealed chronic granulomatous inflammation with extensive necrosis in the left upper lung. **(C)** CT showed the soft - tissue mass in the left upper lung had increased (December 19, 2023); Bronchoscopy revealed a neoplastic lesion infiltrating and narrowing the lumen; Pathological examination confirmed a diagnosis of SCLC. **(D)** CT demonstrated partial resolution of the left upper lung lesion (June 7, 2024). **(E)** CT revealed a new lesion approximately 2.9 cm in the right lower lobe (December 24, 2024). **(F)** CT demonstrated stable lesions in both left upper lobe and right lower lobe (March 23, 2025).

A 68-year-old male presented to the Department of Respiratory Medicine on July 26, 2023, with a six-month history of persistent cough and sputum production, accompanied by exertional chest tightness that had worsened over the previous 10 days. Six months earlier, he was diagnosed with COVID-19 and experienced symptom resolution following treatment. However, post-discharge, his symptoms, including yellow sputum, exertional chest tightness, and fatigue, persisted without fever or chest pain. Intermittent self-administration of anti-inflammatory medications provided no relief. Ten days prior to admission, symptom progression began to limit daily activities. A chest CT conducted on July 22, 2023, revealed left-sided pneumonia and bilateral interstitial lung disease (**Figure 1A**). Treatment with levofloxacin and acetylcysteine was ineffective. The patient, a 40-year smoker (20 cigarettes/day) who had quit three months earlier, underwent blood tests revealing elevated inflammatory markers: White blood cell (WBC) count 9.90×10⁹/L (normal: 3.5–9.5), neutrophil percentage 76.70% (normal: 40–75), and CRP (C-reactive protein) 29.38 mg/L (normal: 0–3). Suspecting a pulmonary infection, the patient was started on intravenous piperacillin-tazobactam (4.5 g every 8 hours) and levofloxacin (3 g once daily), alongside symptomatic treatments.

On July 27, 2023, bronchoscopy revealed diffusely distributed purulent secretions and a submucosal protrusion with hyperemia at the opening of the left upper lobe bronchus (**Figure 1A**). Bronchial lavage and brushing were performed, yielding no malignant cells, while next-generation sequencing of lavage fluid showed no abnormalities. Due to persistent purulent secretions, a second bronchoscopy on August 1, 2023, included a mucosal biopsy of the left upper lobe. Pathological findings revealed mildly dysplastic squamous epithelium, submucosal fibrous tissue proliferation, and mild chronic inflammatory cell infiltration (**Figure 1A**). The patient was discharged on August 2, 2023, with instructions to continue oral levofloxacin and amoxicillin-clavulanate for two weeks.

On August 31, 2023, a follow-up chest CT showed no significant changes compared to prior imaging. As the patient reported no symptom worsening, he was advised to rest at home and continue regular follow-ups. However, on October 5, 2023, contrast-enhanced chest CT revealed persistent irregular bronchial dilations in the anterior segment of the left upper lobe, with significant wall thickening, soft tissue shadows, and uneven enhancement. Multiple small mediastinal lymph nodes were also observed, with no notable changes since the last imaging (**Figure 1B**). Given the persistence of pulmonary lesions, the patient was admitted to the Department of Thoracic Surgery on October 9, 2023. Blood tests showed a WBC count of 9.44×10⁹/L, neutrophil percentage of 70.0%, and CRP level of 15.95 mg/L. Tumor marker levels showed an elevated progastrin-releasing peptide (ProGRP, 127.28 pg/mL; normal ≤67.42), while neuron-specific enolase (NSE, 15.81 ng/mL; normal ≤16.5) and carcinoembryonic antigen (CEA, 2.90 ng/mL; normal ≤4.5) remained within normal ranges. On October 11, 2023, a CT-guided percutaneous lung biopsy was performed (**Figure 1B**), revealing chronic granulomatous inflammation with extensive necrosis, suggestive of tuberculosis. Additionally, focal atypical adenomatous hyperplasia of the alveolar epithelium and nodular hyperplasia of neuroendocrine cells were observed. Special stains for acid-fast bacilli and Alcian blue were negative (**Figure 1B**). Following consultation with infectious disease specialists, multiple sputum smear tests confirmed the presence of acid-fast bacilli. From October 20 to December 19, 2023, the patient received a two-month course of combination anti-TB therapy, including ethambutol, pyrazinamide, rifampicin, and isoniazid (four tablets daily).

The patient experienced worsening symptoms, including increased cough, sputum production, and chest tightness, and was readmitted to the Department of Respiratory Medicine on December 19, 2023. A follow-up contrast-enhanced chest CT revealed an enlarged soft tissue mass in the left upper pulmonary hilum, along with multiple slightly enlarged mediastinal and left pulmonary hilar lymph nodes (**Figure 1C**). Tumor marker tests showed markedly elevated ProGRP levels (513.50 pg/mL; normal ≤67.42), with NSE (29.36 ng/mL; normal ≤16.5) and CEA (5.47 ng/mL; normal ≤4.5) also increased. Lung cancer was strongly suspected based on imaging and tumor marker results. After discussing the findings with the patient’s family, a third bronchoscopy was performed on December 20, 2023, revealing a neoplastic lesion infiltrating and narrowing the left upper lobe bronchus, along with viscous secretions in the lumen. Biopsy and cytologic brushing confirmed a diagnosis of SCLC in the left upper lobe (**Figure 1C**). PET-CT indicated central-type lung cancer with distal obstructive pneumonia, elevated FDG metabolism, and probable left paratracheal lymph node metastasis. The patient was staged as cT3N2M0 (stage IIIB, limited stage). Treatment included four cycles of chemotherapy (etoposide 0.15 g on days 1–3 and cisplatin 40 mg on days 1–3, every 3 weeks) followed by 30 sessions of chest radiotherapy. Despite grade 1–2 adverse reactions, the patient tolerated treatment well. Anti-tuberculosis therapy was temporarily paused during each chemotherapy cycle and resumed afterward, allowing completion of the 11-month course. A follow-up chest CT on June 7, 2024, showed partial resolution of the left upper lung lesion (**Figure 1D**), and tumor markers returned to near-normal levels (ProGRP, 66.20 pg/mL; NSE, 20.10 ng/mL).

On December 24, 2024, the patient reported no significant discomfort at a follow-up visit. However, chest CT revealed a new lesion, approximately 2.9 cm in size, in the right lower lobe, suspected to be metastatic (**Figure 1E**). The left lung lesion remained stable. Tumor markers were again elevated (ProGRP, 219.00 pg/mL; NSE, 27.80 ng/mL), confirming disease progression. The patient was readmitted and started three cycles of chemotherapy with etoposide 0.10 g on days 1–3 and cisplatin 40 mg on days 1–3. On March 23, 2025, repeat chest CT demonstrated improvement in the right lower lobe pulmonary lesion measuring 1.7 cm compared with prior imaging. Tumor markers: ProGRP 155.38 pg/mL; NSE 11.4 ng/mL. Patient assessed as having Stable Disease. Initiated oral anlotinib 10mg once daily on a schedule of 2 weeks on/1 week off. We will continue to monitor disease status and subsequent management.

## Discussion

Since Bayle’s first documented case of pulmonary TB with concurrent lung cancer in 1810, this dual pathology has gained increasing clinical recognition. Epidemiological studies indicate: TB patients exhibit 1.5–11 fold higher lung cancer incidence versus non-TB populations; 1%-2% of TB patients develop lung cancer comorbidity; 2%-4% of lung cancer patients present with active TB coinfection. Co-occurrence rates reach 10%-15% among individuals over 60 (7, 8). The coexistence of lung cancer and active pulmonary TB presents significant diagnostic and therapeutic challenges. In such cases, lung cancer is often diagnosed at advanced stages, contributing to poor prognosis (9). Early detection and timely management are critical to improving outcomes, and preoperative anti-TB therapy for approximately one month may facilitate safer surgical interventions (10, 11). While various histological subtypes of lung cancer, such as adenocarcinoma and squamous cell carcinoma, have been reported in combination with TB, the coexistence of active pulmonary TB and SCLC is exceedingly rare, with limited documentation in the literature (12).

Arliny et al. reported a 54-year-old male with concurrent pulmonary TB and SCLC. Initial chest X-ray findings showed infiltration and a cavity in the right upper lung, leading to a TB diagnosis. After two months of anti-TB therapy, radiological improvement was observed, but clinical symptoms persisted. Bronchoscopy and forceps biopsy subsequently confirmed SCLC (13). Yonemori et al. described a Japanese male patient with SCLC and concurrent Mycobacterium tuberculosis infection, emphasizing the drug interactions between irinotecan and rifampin in extensive-stage SCLC (14). Similarly, Li et al. documented a 54-year-old male presenting with cough and blood-tinged sputum, where bronchoscopic biopsy confirmed SCLC and acid-fast staining identified Mycobacterium tuberculosis (15).

In comparison, the diagnostic process in our case demonstrated greater complexity. During the initial two bronchoscopic examinations, the patient was in the early stages of both TB and SCLC. Extensive purulent secretions were present, but no tumor mass was visible, and mucosal biopsy, brushing, and bronchoalveolar lavage failed to identify tumor cells or Mycobacterium tuberculosis. After two months of anti-infective therapy, the pulmonary lesions showed no improvement. Tumor marker testing revealed elevated ProGRP levels (127.28 pg/mL), prompting a CT-guided biopsy, which identified chronic granulomatous inflammation with massive necrosis, raising suspicion of TB. Consultations and multiple sputum smear tests eventually confirmed active pulmonary TB. However, despite standardized anti-TB treatment, pulmonary lesions continued to progress. A third bronchoscopy revealed neoplastic infiltration at the opening of the left upper lobe, and pathological examination confirmed SCLC.

This patient underwent a prolonged diagnostic process spanning four months, involving three bronchoscopic examinations and one CT-guided biopsy, before a final diagnosis of active pulmonary TB complicated by SCLC was established. Several limitations in the diagnostic approach were noted: 1. Tumor marker testing delay: ProGRP testing was not performed during the initial evaluation. Earlier detection of elevated ProGRP levels might have expedited lung cancer suspicion. 2. Missed opportunities for multidisciplinary consultation: Despite no improvement in pulmonary lesions after one month of anti-infective therapy, a multidisciplinary consultation was not promptly conducted. Such consultations could have integrated multiple perspectives, minimizing the risk of missed diagnoses. 3. Delayed PET-CT examination: When biopsy results were inconclusive, PET-CT was not immediately recommended. This imaging modality could have provided critical information about the metabolic activity of pulmonary lesions, facilitating earlier identification of SCLC. A more systematic diagnostic approach, integrating tumor marker testing, advanced imaging, and multidisciplinary consultations, might have resulted in earlier and more accurate diagnosis, enabling timely treatment.

Both TB and SCLC commonly present with non-specific symptoms, such as persistent cough, hemoptysis, chest pain, fever, weight loss, and night sweats, complicating differentiation based on clinical features alone (16, 17). Similarly, imaging findings—including lung consolidations, nodules, cavitations, and lymphadenopathy—are often non-specific and can be attributed to either condition (18, 19). In regions with a high TB prevalence, this overlap often leads to diagnostic delays, as initial investigations prioritize TB over malignancy. Bronchoscopy is a valuable diagnostic tool, enabling direct airway visualization and tissue sampling. However, its sensitivity for TB diagnosis is limited, particularly when the infection is not localized or the bacterial load is low, leading to false-negative results. For SCLC, bronchoscopy often plays a critical role in obtaining histopathological confirmation, although factors such as tumor location or submucosal infiltration may reduce its diagnostic yield. CT-guided percutaneous biopsy provides another essential modality, particularly for peripheral lesions and mediastinal abnormalities. Despite its higher diagnostic accuracy, limitations include risks such as pneumothorax and bleeding, as well as compromised yield in cases of necrotic or insufficient tissue (20–22). In this case, the diagnostic complexity necessitated repeated procedures, including bronchoscopy and CT-guided biopsy, to accurately identify active TB and SCLC. These repeated evaluations underscore the importance of maintaining a high index of suspicion, carefully interpreting clinical and imaging findings, and strategically planning diagnostic techniques. Optimizing diagnostic workflows requires a multidisciplinary approach. Collaboration among pulmonologists, thoracic surgeons, oncologists, radiologists, and pathologists facilitates the integration of clinical, radiological, and pathological data. This approach minimizes misdiagnoses and ensures timely treatment initiation, addressing the dual challenges of TB and malignancy effectively.

The concurrent management of active pulmonary TB and SCLC presents unique therapeutic challenges, including potential drug-drug interactions, overlapping toxicities, and the need to balance effective treatment of both conditions. Standard anti-TB therapy typically comprises isoniazid, rifampin, pyrazinamide, and ethambutol (23). Isoniazid and rifampin may reduce white blood cell and platelet counts, and cause liver damage-adverse reactions also common with antitumor chemotherapy. Therefore, managing pulmonary tuberculosis with lung cancer requires consideration of multiple drug interactions and overlapping toxicities. In addition, rifampin, a potent inducer of the cytochrome P450 enzyme system, significantly alters the metabolism of various drugs, including chemotherapeutic agents such as cisplatin and etoposide, thereby reducing their efficacy (24–26). Hepatotoxicity is a common adverse effect of both anti-TB drugs and chemotherapy, increasing the risk of cumulative liver damage. Close monitoring of liver function tests is essential to detect hepatotoxicity early and prevent complications (27–29). In cases of significant hepatic impairment, dose adjustments or temporary cessation of therapy may be required. Alternatively, chemotherapeutic agents with lower hepatic metabolism can be considered to mitigate liver toxicity. Current evidence suggests initiating chemotherapy 2–3 weeks after standard quadruple antituberculosis therapy is relatively safe. This timing allows rapid reduction of Mycobacterium tuberculosis burden and sputum bacilli clearance, minimizing chemotherapy risks (30). Concurrent chemotherapy and antituberculosis treatment has also demonstrated safety in clinical studies (4, 5). Given the aggressive nature of SCLC, timely initiation of chemotherapy is critical for improving survival. However, concurrent anti-TB therapy is necessary to prevent progression of infection and related complications. A multidisciplinary approach is vital to develop individualized treatment plans based on disease severity and progression. Sequential or staggered therapy may be beneficial when significant drug interactions or toxicities are anticipated. For instance, initiating anti-TB therapy for a few weeks to stabilize infection before starting chemotherapy can minimize TB-related immunosuppression (5, 31). For patients with minimal systemic TB involvement, simultaneous administration of both therapies under close monitoring may be feasible (6). Supportive care measures, including growth factor support, nutritional optimization, and infection control, are crucial for maintaining overall health during combined treatment. These interventions help mitigate treatment-related adverse effects and improve therapy tolerability.

In this case, the patient received two months of anti-TB therapy before the diagnosis of SCLC. This initial treatment likely played a critical role in stabilizing TB, facilitating the subsequent administration of chemotherapy. To minimize drug interactions and side effects, anti-TB drugs were temporarily paused for one week during each chemotherapy cycle. The patient completed a total of 11 months of anti-TB treatment, achieving stable TB control. At the most recent follow-up, nearly five months after completing anti-TB therapy, the patient’s pulmonary TB remained stable. This case underscores the importance of individualized treatment planning for coexisting TB and SCLC. By carefully coordinating anti-TB therapy and chemotherapy and implementing supportive care strategies, both conditions were effectively managed without significant adverse events. Key recommendations for future management: 1. Comprehensive Baseline Assessment: Perform thorough evaluations, including liver and renal function tests and imaging studies, to identify potential complications and guide treatment planning. 2. Integrated Multidisciplinary Approach: Collaboration among pulmonologists, oncologists, infectious disease specialists, and pharmacists is crucial for optimizing treatment strategies and ensuring close monitoring. 3. Tailored Therapy: Personalize treatment based on disease severity, comorbidities, and the risk of drug interactions or toxicities. Decisions regarding simultaneous or sequential therapy should consider the patient’s overall condition and treatment tolerance. 4. Rigorous Monitoring: Regular follow-up with clinical assessments and laboratory tests is essential for early detection and management of adverse effects, particularly hepatotoxicity. 5. Research and Evidence Building: Documenting and analyzing similar cases can improve understanding of this dual pathology and contribute to evidence-based guidelines for managing TB and SCLC. The concurrent management of TB and SCLC requires a balanced, patient-centered approach. This case highlights the complexities of treating these conditions and emphasizes the importance of early diagnosis, strategic treatment planning, and multidisciplinary collaboration. Insights from cases like this can inform clinical practice and improve outcomes for patients facing similar challenges.

The simultaneous presence of TB and lung cancer may result from distinct but interconnected biological mechanisms. Chronic inflammation and immune dysregulation induced by TB infection can promote tumorigenesis, while the immunosuppressive environment created by lung cancer or its treatments predisposes patients to latent TB reactivation (32–34). Pulmonary TB is driven by persistent inflammation, involving pro-inflammatory cytokines such as tumor necrosis factor-alpha, interleukin-6 and interferon-gamma. Chronic inflammation leads to genomic instability and DNA damage, fostering a microenvironment conducive to tumorigenesis (35, 36). Reactive oxygen species and reactive nitrogen species generated during TB infection further exacerbate DNA damage, promoting carcinogenesis (37). TB-associated immune dysfunction also impairs immune surveillance, enabling cancer cells to evade immune control. Mycobacterium tuberculosis modulates macrophage and T-cell function, potentially suppressing anti-tumor immune responses and facilitating tumor progression (38–40). Granulomatous inflammation, a hallmark of TB, promotes angiogenesis through vascular endothelial growth factor upregulation. Enhanced angiogenesis supports TB progression and simultaneously creates a microenvironment favorable for tumor growth and metastasis (41). Additionally, both TB and SCLC have been associated with epigenetic modifications, such as DNA methylation and histone acetylation, which influence gene expression and may predispose lung epithelial cells to malignant transformation during chronic TB infection (42). TB-associated proteins may also activate cellular signaling pathways, including the nuclear factor-kappa B pathway, which plays a key role in inflammation and cancer development (43, 44). Latent TB infection may persist in lung tissues, acting as a chronic irritant and potential trigger for carcinogenesis over time (32, 45).

These complex biological interactions highlight the importance of understanding the interplay between TB and SCLC for advancing diagnostic and therapeutic strategies. Insights into this relationship could lead to the identification of biomarkers for early detection, targeted therapies to mitigate TB’s tumor-promoting effects, and preventive measures to reduce cancer risk in TB patients. Further molecular and epidemiological research is needed to elucidate the precise mechanisms underlying this association. Such studies could provide valuable insights into the pathogenesis of TB-associated malignancies and inform integrated disease management approaches.

## Summary

The coexistence of active pulmonary TB and SCLC represents an exceptionally rare clinical scenario that poses significant diagnostic and therapeutic challenges. Overlapping clinical and radiological features often result in diagnostic delays, requiring a high index of suspicion, particularly in TB-endemic regions. When initial evaluations are inconclusive, repeated and multimodal diagnostic approaches, including tumor marker assessments, bronchoscopy, advanced imaging, and tissue biopsies, are critical for establishing an accurate diagnosis. Effective management of concurrent TB and SCLC requires meticulous planning and close multidisciplinary collaboration among pulmonologists, oncologists, infectious disease specialists, radiologists, pathologists, and pharmacists. This approach optimizes diagnostic accuracy, balances drug interactions and overlapping toxicities, and ensures individualized treatment strategies. Given the rarity of this dual pathology, further case reports and research are vital to advancing understanding of the biological interplay between TB and SCLC. These efforts can refine diagnostic protocols, establish evidence-based treatment guidelines, and improve patient outcomes. Clinicians are encouraged to contribute similar cases to expand the knowledge base and enhance care for this clinically significant yet rare combination of diseases.

## Data Availability

The original contributions presented in the study are included in the article/supplementary material, Further inquiries can be directed to the corresponding author/s.
